# Outcome prediction for hypothermic patients in cardiac arrest

**DOI:** 10.1186/s40560-022-00630-7

**Published:** 2022-07-28

**Authors:** Mathieu Pasquier, Olivier Hugli, Nicolas Hall, Valentin Rousson, Tomasz Darocha

**Affiliations:** 1 Department of Emergency Medicine, Lausanne University Hospital and University of Lausanne, Lausanne, Switzerland; 2 Institute of Social and Preventive Medicine, Unisanté, route de la Corniche 10, 1010 Lausanne, Switzerland; 3 Severe Accidental Hypothermia Center, Department of Anaesthesiology and Intensive Care, Medical University of Silesia, Katowice, Poland

## Abstract

The 5A score predicts in-hospital mortality of patients suffering from accidental hypothermia, including those not in cardiac arrest. The HOPE score was specifically developed to predict survival for the subgroup of hypothermic patients in cardiac considered for extracorporeal life support rewarming. The C-statistic in the external validation study of the HOPE score was 0.825 (95% CI: 0.753–0.897), confirming its excellent discrimination. In addition, its good calibration allows for a reliable interpretation of the corresponding survival probability after rewarming. The HOPE score should be used for predicting outcome and selecting hypothermic patients in cardiac arrest for rewarming.

Dear Editor,

We read with great interest the article on the external validation of the 5A score to predict in-hospital mortality of accidental hypothermia patients and published by Okada et al. [1]. We would like to comment on some of their claims made about two studies the we recently published and pertaining to the HOPE (Hypothermia Outcome Prediction after ECLS rewarming) score, which predicts survival for hypothermic patients in cardiac arrest if offered extracorporeal life support (ECLS) rewarming [2].

As a first strength, the authors stated that the 5A score was the first externally validated prediction model for use in patients with accidental hypothermia. It is true that in general, predictive models are rarely subject to external validation studies, and the authors are to be commended for the validation of their 5A score [3]. However, their statement is incorrect. The HOPE score was not only internally validated using bootstrapping, but also by an external validation study with 122 patients, including 49 further additional unpublished hospital cases [2, 4]. Contrary to the authors’ claim, the HOPE score was not only developed using patients reported in case report format, but also included 49 unpublished hospital cases. Furthermore, like in the study of Okada et al., we included only studies with consecutive patients, in order to minimize the risk of inclusion bias.

The C-statistic obtained in the validation study for the 5A score was 0.736 (95% CI: 0.699–0.772). It was inferior to the value reported in the external validation study of the HOPE score: 0.825 (95% CI: 0.753–0.897), confirming the HOPE score excellent discrimination. The calibration of the HOPE score in the validation study was also good, allowing for a reliable interpretation of the corresponding HOPE in-hospital survival probability after ECLS rewarming. No additional categorization of mortality risk is required, unlike in the interpretation of the 5A score. The HOPE score is now used in clinical practice, to select patients in hypothermic cardiac arrest at hospital admission for further resuscitation by ECLS methods. Its validity has been acknowledged, and it is now part of the decision algorithm for cardiac arrest due to accidental hypothermia published in the 2021 ERC guidelines [5].

The derivation study of the 5A score included 74/532 (13.9%) patients with "Near cardiac arrest (CA)" and only 20/532 (3.8%) who underwent VA-ECMO [6]. The latter proportion is far smaller than in the HOPE derivation study, which included 286 patients undergoing ECLS rewarming [2]. In addition, only 0.5% of patients had outdoor exposure as the primary cause of hypothermia in the validation study of the 5A score, while 74% presented with hypothermia in an indoor setting. These figures suggest an underlying medical conditions as the triggering event leading to hypothermia [1]. Patients’ body temperatures in the 5A study ranged between 28 and 32.7 °C. In the external validation of HOPE, patients’ core temperatures were much lower, between 22 and 27 °C. Their hypothermia was secondary to outdoor exposure, avalanche accident, immersion or submersion in most cases. The populations therefore differed significantly between the two scores, thus the respective scores must be applied to patients similar to those from which the scores were derived.

The outcomes were also different: overall in-hospital mortality for mildly hypothermic patients for the 5A score and in-hospital mortality after ECLS rewarming for severely hypothermic in cardiac arrest for the HOPE score. The high-risk in-hospital mortality subgroup included in the derivation study of the 5A score represents precisely the target group for the HOPE score.

Clinicians may use the 5A and the HOPE scores as complementary rather than exclusive, as they clearly target different populations. In the specific case of hypothermic CA, the HOPE score should be used for predicting in-hospital mortality following ECLS rewarming.

## Data Availability

Not relevant.
